# Association Study of *KCNH7* Polymorphisms and Individual Responses to Risperidone Treatment in Schizophrenia

**DOI:** 10.3389/fpsyt.2019.00633

**Published:** 2019-08-30

**Authors:** Xueping Wang, Yi Su, Hao Yan, Zhuo Huang, Yu Huang, Weihua Yue

**Affiliations:** ^1^Peking University Sixth Hospital, Institute of Mental Health, Beijing, China; ^2^NHC Key Laboratory of Mental Health, Peking University, Beijing, China; ^3^National Clinical Research Center for Mental Disorders, Peking University Sixth Hospital, Beijing, China; ^4^State Key Laboratory of Natural and Biomimetic Drugs, Department of Molecular and Cellular Pharmacology, School of Pharmaceutical Sciences, Peking University Health Science Center, Beijing, China; ^5^Key Laboratory for Neuroscience, Ministry of Education, Beijing, China; ^6^National Engineering Research Center for Software Engineering, Peking University, Beijing, China

**Keywords:** schizophrenia, risperidone medications, KCNH7, treatment responses, pharmacogenomics

## Abstract

Risperidone has been used to treat the symptoms of schizophrenia and to reduce its relapse. However, the responses to treatment show great variability among patients. The potassium channel has been reported as an effective target for antipsychotics. *KCNH7*, a member of the voltage-gated K^+^ channel Kv11 family, is primarily expressed in the brain. Here, we assessed the genetic association of *KCNH7* with risperidone responses in 393 schizophrenia patients. The patients were treated with risperidone for 6 weeks. The reduction rates of Positive and Negative Syndrome Scale (PANSS) scores were determined to quantify drug response. We also examined the associations between six single-nucleotide polymorphisms (SNPs) of *KCNH7* and the risperidone responses for a total of 6 weeks. The SNP rs77699177 (C > T) in the *KCNH7* gene intron was significantly associated with the treatment response reflected by the PANSS reduction rate (CC, 55.8 ± 23.0; TC, 70.9 ± 20.3, *P* = 0.000110), indicating that patients with the TC genotype have better efficacy for antipsychotic therapy. The rs2241240 SNP also showed a significant association with treatment responses after 6 weeks of treatment (*P* = 0.00256). The findings indicate that the voltage-gated K^+^ channel *KCNH7* is a potential functional marker for the identification of the response to risperidone treatment in schizophrenia patients.

Note: The study was registered under clinical trial number ChiCTR-RNC-09000522 (http://www.chictr.org/).

## Introduction

Schizophrenia is a severe psychiatric disorder characterized by both positive and negative symptoms. Typical symptoms of schizophrenia include hallucinations, delusions, thought disorders, avolition, affective flattening, and social withdrawal. The condition affects about 1% of the population worldwide (1), and patients require treatment throughout their lives because of the chronic and debilitating course of this disease. Thus, schizophrenia imposes a heavy burden on both individual patients and the public health-care system (2).

Clinical management of schizophrenia involves different antipsychotic drugs, including typical/first-generation antipsychotics (FGAs) and atypical/second-generation antipsychotics (SGAs). These drugs are known to improve positive and negative symptoms in many patients. However, responses to drug treatment show wide variability (3). A previous review reported that approximately 75% of patients exhibit low drug compliance because of ineffectiveness or side effects of these drugs (4), consequently leading to relapse, clinical exacerbation, and/or repeated admissions. Therefore, understanding the individual responses to antipsychotics is imperative for the development of precision medicine, and it is critical to establish a personalized antipsychotic treatment system to improve the clinical outcome.

Studies have suggested that genetic factors play a significant role in personal responses to medication (5–7), and many genetic variants of the dopamine and serotonin receptor genes have been reported in the past decades (8–12). The Kv11 family, also called the HERG family, belongs to the subfamily H of the voltage-gated potassium (Kv) channels and plays an important role in the membrane potential regulation (13). The Kv11 family includes three members, which are *KCNH2*/*Kv11.1*, *KCNH6*/*Kv11.2*, and *KCNH7*/*Kv11.3* (14). All Kv11 channels are expressed in the nervous system. *KCNH7* and *KCNH2* are expressed in the brain (15, 16), and *KCNH6* is predominantly expressed in the superior mesenteric ganglia (15). These expression data indicate their functions in the nervous system. Several studies have reported *KCNH2* as a risk gene for schizophrenia (16–19). One recent study revealed that schizophrenia patients with different *KCNH2* genotypes showed different responses to risperidone, a common antipsychotic drug used in the clinic (20). The findings suggest that the Kv11 family plays an important role in the antipsychotic response.

The *KCNH7* gene shows higher expression levels in the brain than in other tissues (15), while the *KCNH2* gene has higher expression levels in the heart than in the brain (16). In this study, we hypothesized that *KCNH7* contributes to the outcomes of risperidone treatment in schizophrenia patients. To this end, we performed a pharmacogenetics association study to identify the genetic function of *KCNH7* and examined the associations between the *KCNH7* genotype and risperidone efficacy in schizophrenia patients of Han Chinese ancestry over a 6-week acute treatment.

## Methods

### Study Design and Participants

A total of 452 patients were enrolled at our hospital and three other hospitals (from Hebei, Henan, and Liaoning provinces), and among these patients, 393 underwent single-nucleotide polymorphism (SNP) genotype assessment. Patients included in this study met the following inclusion criteria: (I) diagnosed with schizophrenia based on the Structured Clinical Interview of the *Diagnostic and Statistical Manual of Mental Disorders*, fourth edition, Text Revision (DSM-IV-TR); (II) aged 18–45 years; (III) a Han Chinese lineage; (IV) first-onset or chronic disease in the acute phase with severe symptoms; (V) total scores of more than 70 on the Positive and Negative Syndrome Scale (PANSS); and (VI) provided written informed consent.

The exclusion criteria were as follows: (I) pregnancy or breast-feeding; (II) contraindications to the recommended drugs; (III) severe or unstable physical diseases; (IV) presence of QTc > 450 ms in males or QTc > 470 ms in females, decompensated and congestive heart failure, or complete left bundle branch block delay; (V) a history of serious suicide attempts or severe excitement and agitation episodes; and (VI) requirement of long-acting injectable medication to maintain treatment adherence or regularly treated with clozapine for treatment over the past month.

We recruited patients and performed several routine baseline assessments at the start of the study, including DSM-IV-TR, inclusion/exclusion criteria selection, patient general information records, and Positive and Negative Syndrome Scale (PANSS). Within the first 2 weeks after enrollment, clinicians could adjust the drug dosages based on the treatment effectiveness (risperidone 2–6 mg/day), and the adjusted dosage of the antipsychotics remained unchanged throughout the rest of the study period. The patients visited the participating clinicians at weeks 2, 4, and 6, and their PANSS scores were recorded by the psychiatrists. All the patients and clinicians were blinded to the study design. Treatment was discontinued if the patient response was not adequate or if the patient decided to withdraw from the study; in that case, the last-observation-carried-forward procedure was used to show her or his treatment response. Patients with adequate responses continued treatment for up to 6 weeks. The objective and procedure of this study were explained to all participants, and all included participants provided written informed consent. The study was approved by the research ethics committees and institutional review boards of each local hospital. All procedures were conducted in accordance with the principles expressed in the Declaration of Helsinki. The study was registered under the clinical trial number ChiCTR-RNC-09000522 (http://www.chictr.org/).

A total of 59 individuals withdrew from this study, and the reasons were as follows: withdrawing the informed consent and refusing to continue treatment according to the study protocol (36 individuals); lost to follow-up (13 individuals); occurrence or recurrence of severe physical diseases during treatment (7 individuals); and changed treatment according to the doctors’ advice (3 individuals).

### Phenotype Definition

Using the percentage changes in the PANSS scores to indicate treatment effects is a common strategy in statistical analyses. The PANSS is an interval scale with each item ranging from 1 to 7 and lacking a natural zero point. The 30-item PANSS yields scores of 30∼210. Categorizations using the PANSS may reduce the sensitivity and power of statistical tests. Therefore, continuous measures are suggested to cutoff-based dichotomous measures of treatment responses. We used percentage changes in the PANSS scores to evaluate acute responses to risperidone medications. The theoretical minimum (30 for the total score) was subtracted from the baseline score to avoid incorrect calculations, leading to a score range including zero (21). The PANSS change from baseline was defined as PANSS endpoint score − PANSS baseline score. The percentage changes of the PANSS reduction rate was defined as follows: the reduction rate of the total score of PANSS = (PANSS baseline score − PANSS endpoint score) ÷ (PANSS baseline score − 30) × 100%. The higher reduction rate indicates a better efficacy for antipsychotic therapy.

### Genotyping

Genomic DNA was extracted using the QIAamp DNA Mini Kit (QIAGEN, Hilden, Germany). Sample genotype sequencing was conducted using the Sequenom MassARRAY system (Sequenom iPLEX assay). The DNA samples from 393 schizophrenia patients were amplified by multiplex polymerase chain reactions (PCRs), and the PCR products were then assessed for locus-specific single-base extension reactions. The primer sequences for PCR and extension are listed in **Table S1**. We selected six SNPs randomly across the gene, with more than 300-kb inter-SNP space, from different linkage disequilibrium (LD) blocks. The pairwise LD analysis was applied to detect the inter-marker relationship, using *D*′ values.

### Statistical Analyses

After quality control, we used linear regression under an additive genetic model to evaluate the associations between the allele dosages and the PANSS percentage changes in PLINK v1.07 (22). We used percentage changes of PANSS to assess the treatment responses to antipsychotic medications in this study. The gender, age, and the first five principal components of the population structure were used as covariates in the analysis. The *P* values were adjusted by multiple comparisons (false discovery rate (FDR)).

The repeated-measures linear mixed model in SPSS (IBM SPSS Statistics 20) was used to analyze the assessments at different time points. The longitudinal PANSS percentage change was analyzed using linear mixed models in SPSS. We classified the subject as a “Subjects” variable and WEEK as a “Repeat” variable. The first-order autoregressive [AR(1)] covariance structure was selected, and we then defined the reduced PANSS percentage as a dependent variable, the genotype of *KCNH7* as a categorical factor, and the WEEK as a continuous covariate. In the model, fixed effects involved WEEK, genotype, and the interaction between WEEK and genotype, and the Subject was defined as a random effect.

For the associated SNPs, we examined their genome-wide cis-e-quantitative trait loci (eQTL) effects in the Brain Expression Consortium (Braineac, GTEx, and Blood eQTL).

## Results

### PANSS Reduction Rate Indicates the Response to Risperidone Treatment

A total of 393 schizophrenia patients remained after quality control. All patients had Han Chinese ancestry and were unrelated to each other. We collected basic information of the included patients and performed baseline assessments at the start of the study. The mean age at study entry was 31.6 ± 8.0 years, and the baseline PANSS score was 89.3 ± 15.3, as shown in **Table 1**. The patients received risperidone monotherapy, and the PANSS scores were assessed after 2, 4, and 6 weeks to reflect the treatment effect. The endpoint PANSS score after the 6-week risperidone monotherapy was 55.4 ± 15.4, which indicated the general treatment effect of risperidone. The PANSS reduction rate was 57.2 ± 23.1, as shown in **Table 1**.

**Table 1:** Demographic and clinical characteristics of 393 patients following 6-week risperidone monotherapy.

Index	Mean ± SD or *n* (%)
**Age at study entry, years**	31.6 ± 8.0
**Gender, n (%)**	
Men/women	227 (57.8)/166 (42.2)
Clinical assessments*	
Baseline PANSS total score	89.3 ± 15.3
Endpoint PANSS total score	55.4 ± 15.4
PANSS reduction rate (%)	57.2 ± 23.1

*Scores on the Positive and Negative Syndrome Scale (PANSS) for schizophrenia can range from 30 to 210, with higher scores indicating more severe psychopathology. The 30-item PANSS measures a broad range of symptoms typically observed in schizophrenia.

### *KCNH7* Shows a Significant Association With Treatment Responses

We next analyzed the association between SNPs of *KCNH7* and the PANSS reduction rate. Linear regression analysis revealed that SNPs showed differential associations with treatment responses (**Table 2**). The most significant association was observed between treatment responses and SNP rs77699177 at the intron regions of the *KCNH7* gene. The PANSS reduction rates were statistically significant in patients with rs77699177 after treatment for 6 weeks (CC, 55.8 ± 23.0; TC, 70.9 ± 20.3, *P* = 0.000110), indicating that patients with the TC genotype have better efficacy for risperidone therapy. In addition, the PANSS reduction rates were also statistically significant in patients with rs2241240 (TT, 58.7 ± 22.3; CT + CC, 50.9 ± 25.2, *P* = 0.00256). However, other SNPs did not show significant associations with treatment responses.

**Table 2:** The association of *KCNH7* gene polymorphisms with risperidone treatment responses after 6 weeks.

SNP	Position	Location	Genotype	*N* (freq.)	Baseline PANSS score Mean ± SD	Change from baseline	PANSS reduction rate (%)	Beta	SE	P*	*P*. adjusted (FDR)
						Mean ± SD	Mean ± SD				
rs77699177	chr2:162476371	Intron	CC	359 (0.913)	89.1 ± 15.2	−33.0 ± 16.8	55.8 ± 23.0	−15.89	4.083	0.000110	0.000657
			TC	34 (0.087)	91.8 ± 16.2	−43.1 ± 15.0	70.9 ± 20.3				
			TT	0							
rs2241240	chr2:162422815	Intron	TT	314 (0.799)	89.3 ± 14.8	−34.7 ± 16.1	58.7 ± 22.3	8.561	2.829	0.00256	0.00769
			CT + CC	79 (0.201)	89.4 ± 17.4	−30.6 ± 19.3	50.9 ± 25.2				
rs12991788	chr2:162731734	Intron	AA	319 (0.812)	89.3 ± 15.5	−33.3 ± 16.5	56.3 ± 22.7	−4.848	2.796	0.0826	0.165
			GA + GG	74 (0.188)	89.5 ± 14.8	−36.3 ± 18.1	60.8 ± 24.5				
rs13404874	chr2:162539839	Intron	GG	236 (0.600)	89.4 ± 15.4	−34.8 ± 16.7	58.9 ± 22.5				
			AG	139 (0.354)	88.3 ± 14.7	−32.1 ± 16.9	54.6 ± 24.0	3.129	1.999	0.116	0.174
			AA	18 (0.046)	96.7 ± 17.4	−35.9 ± 18.3	54.1 ± 23.7				
rs16846992	chr2:162611723	Intron	AA	288 (0.733)	89.8 ± 15.3	−34.5 ± 17.1	57.8 ± 23.2				
			GA	94 (0.239)	87.8 ± 15.2	−31.9 ± 16.0	55.0 ± 23.2	1.257	2.271	0.577	0.693
			GG	11 (0.028)	90.0 ± 17.4	−35.1 ± 15.6	58.9 ± 21.5				
rs1017406	chr2:162786897	Intron	CC	104 (0.265)	88.2 ± 14.2	−34.1 ± 15.4	58.7 ± 23.1				
			CA	191 (0.486)	90.5 ± 15.8	−34.2 ± 17.8	56.2 ± 22.8	0.5439	1.629	0.741	0.741
			AA	98 (0.249)	88.3 ± 15.4	−33.1± 16.5	57.3 ± 23.8				

SNP, single-nucleotide polymorphism; SE, standard error.

*P values are for the association between the presence of SNPs and a response to risperidone.

The minor alleles of rs2241240 and rs12991788 in this study were less than 4; thus, they were incorporated into heterozygous genotype in the analyses.

**Figure 1:**
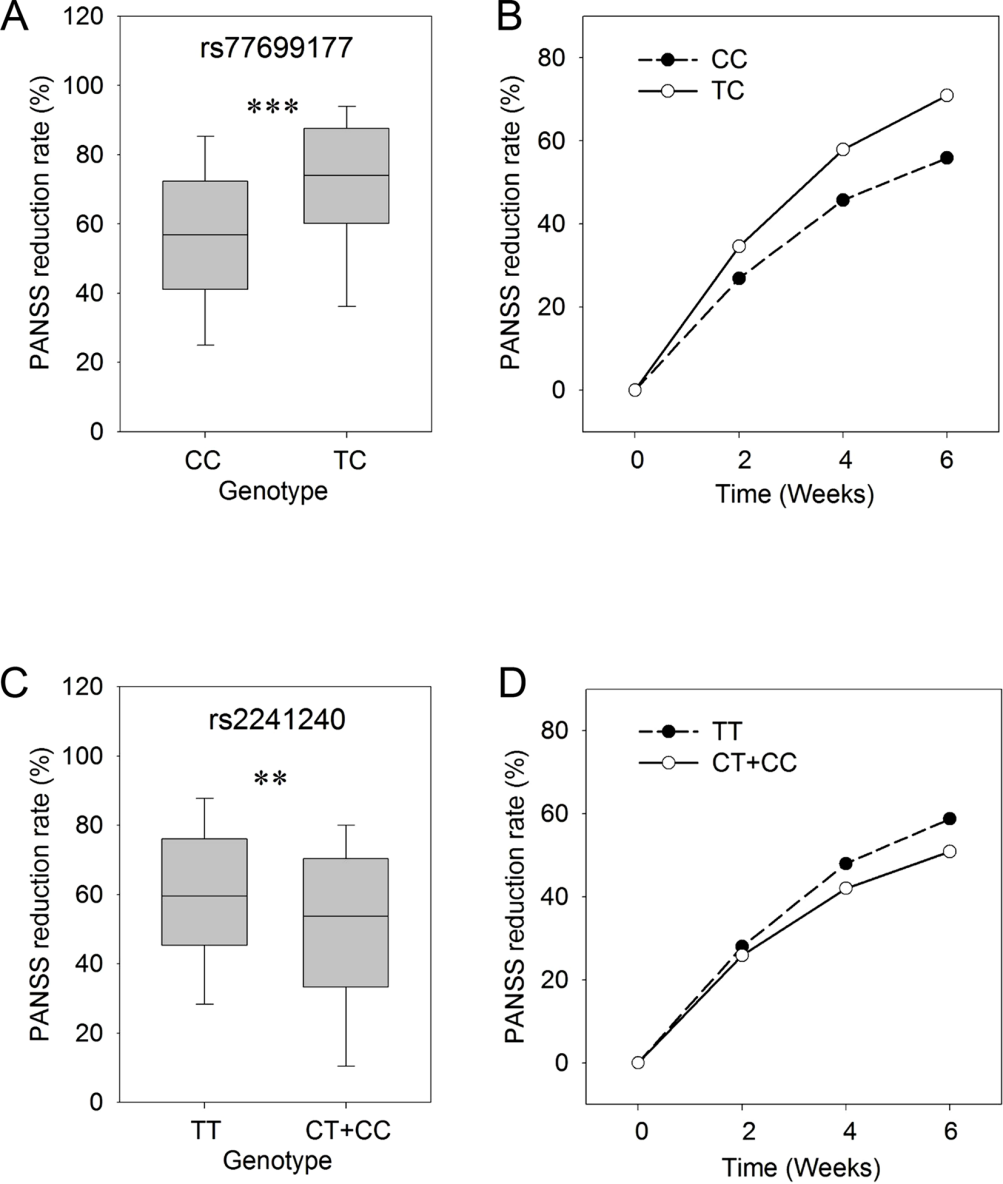
The genotypes of rs77699177 and rs2241240 associated with PANSS reduction rates following risperidone monotherapy. Box plot of the KCNH7 rs77699177 CC/TC allele and the PANSS reduction rates after 6 weeks **(A)**. The PANSS reduction rates during the 6-week risperidone treatment **(B)**. Plot of the rs2241240 TT/(CT + CC) allele after 6 weeks **(C)** and the change over 6 weeks **(D)**. **P < 0.001; ***P < 0.0001.

In order to study the time course of responses in the treatment process, we assessed the PANSS reduction rates at 2, 4, and 6 weeks for each of the SNP genotypes. In this analysis, the PANSS reduction rates in patients with different genotypes (CC/TC) for rs77699177 showed significant differences (*P* ≤ 0.001) after 6 weeks of risperidone treatment (**Figure 1A**). The patients carrying the TC genotype showed better responses to the risperidone treatment for a total of 6 weeks. Significant differences in treatment responses were observed between the two sets of rs77699177 throughout the treatment process (*P* = 0.000569) (**Figure 1B**). In addition, significant PANSS percentage changes were found in patients with rs2241240 after the 6-week treatment (*P* ≤ 0.01) (**Figure 1C**), and the difference between the two sets during the 6-week treatment was noticeable (*P* = 0.0317) (**Figure 1D**). Therefore, SNP rs77699177 may serve as a genetic indicator for risperidone usage.

Among the three members of the Kv11 family (*KCNH2*, *KCNH6*, and *KCNH7*) (14), the *KCNH7* gene shows the highest expression level in the brain (**Figure S1**, https://www.ncbi.nlm.nih.gov/gene) and has a high expression during embryonic development (**Figure S2**, http://hbatlas.org/pages/hbtd). We attempted to assess the expression quantitative trait loci (eQTL) in several databases (Braineac, GTEx, and Blood eQTL). Unfortunately, the SNPs rs77699177 and rs2241240 analyzed in this study were not included in the eQTL databases. The eQTL for *KCNH7* SNPs in several brain tissues is listed in **Table S2**, which was derived from the Braineac database (23). Both rs918927 and rs2216897 showed significant eQTL effects in the cerebellar cortex (**Figure S3**, http://www.braineac.org).

## Discussion

Proper and optimal use of antipsychotic drugs personalized to each patient is important in psychiatric therapy. However, limited evidence is available to guide the use of antipsychotic drugs. Our findings showed that a SNP in *KCNH7* (rs77699177; CC, 55.8 ± 23.0; TC, 70.9 ± 20.3, *P* = 0.000110) was associated with response to risperidone treatment, and patients with the TC genotype had better efficacy for risperidone therapy. *KCNH7* encodes HERG3/Kv11.3, which is involved in potassium homeostasis and is essential for neuronal excitability. The present study indicates that rs77699177 in the *KCNH7* gene could serve as a genetic factor in the identification of the treatment response to risperidone. These findings provide further evidence for the neuronal function of the Kv channel in the pathology of schizophrenia.

Risperidone is used to treat not only schizophrenia but also bipolar disorder and irritability associated with autism (24–26). Studies have demonstrated the association between *KCNH7* and bipolar disorder and autism (27–29). A non-synonymous variant of *KCNH7* (c.1181G > A, p.Arg394His), rs78247304, has a strong family-based association with bipolar spectrum disorder (27). Targeted massively parallel sequencing demonstrates a nominal gene-based rare variant association between autism spectrum disorder and *KCNH7* (*P* = 0.043) (29). *KCNH7* might be an important genetic factor in the efficacy of risperidone against mental disorders. Further studies are needed to investigate the pharmacogenetic relationships between *KCNH7* and risperidone in bipolar disorder and autism, and the possible role of *KCNH7* in the treatment efficacy of risperidone in bipolar disorder remains to be investigated.

Pharmacogenetic studies have investigated the genetic variants of neurotransmitter receptors, such as the dopamine and serotonin receptors (5). A number of clinical studies have examined the dopamine receptors (*DRD2*, *DRD3*, and *DRD4*), serotonin receptors (*HTR2A*, *HTR2C*, *5-HT6*, and *5HTT*), and genes related to their metabolic pathways (such as *COMT and* *CYP2D6*) (5). However, many genetic variants display variable results, probably because of the limited effect size. The K^+^ channel *KCNH2* is now being assessed in pharmacogenetic studies. The primate-specific isoform *KCNH2*-3.1 affects cognition and neuronal repolarization and is associated with the risk of schizophrenia (16, 18, 19). Different antipsychotics have a distinct affinity for KCNH2 (30). One recent study found that risperidone caused greater block of KCNH2-3.1 isoform than that of full-length KCNH2 in electrophysiology recordings (20). The clinical observations suggest that patients with the *KCNH2* risk genotype (higher *KCNH2*-3.1 expression levels) combined with slow metabolism status have better responses to risperidone treatment (20). The findings mentioned above further support the theory that the Kv11 channel could be the functional target for risperidone.

Two recent studies showed that potassium channel Kv2.1 and Kv7.2/7.3 could separately form complexes with dopamine transporter and affect dopamine transport and electrophysiological function (31, 32). A previous study found that the haloperidol treatment could block the K^+^-induced dopamine release in the caudate–putamen and nucleus accumbens and prevent K^+^-induced hyperlocomotion (33). However, whether KCNH7 and other members of Kv11 have similar roles remains unclear. In the Kv11 family, the three genes *KCNH7*, *KCNH2*, and *KCNH6* show differentiated expression patterns (**Figure S1**, https://www.ncbi.nlm.nih.gov/gene). *KCNH2* and *KCNH7* show higher expression levels in the brain than *KCNH6* (**Figure S1**), and *KCNH7* has relatively high expression levels in neurons (**Figure S4**, http://www.alzdata.org), suggesting its role in the central nervous system. *KCNH2* and *KCNH7* might have overlapping functions in the central nervous system. In the study by Heide et al., risperidone showed greater blockade for *KCNH2*-3.1 than for *KCNH2*-1A in the electrophysiology assay (20); however, additional assessments were hindered because of the small sample size. Our study included 393 eligible patients in the association analysis, and the responder analysis for SNPs of KCNH7 gene is listed in **Table S3**. The SNPs (rs77699177 and rs2241240) found in our study are in the intron of the *KCNH7* gene, and the specific functional study of the channel protein is thus limited. Moreover, information is lacking on these SNPs in the current public eQTL databases (Braineac, GTEx, and Blood eQTL Browser), probably because of their low minor allele frequencies in people of European descent. The other possible reason is the unchanged expression levels of *KCNH7* between controls and schizophrenia patients (**Figure S5**, http://www.szdb.org/index.html). Thus, the SNPs in *KCNH7* might affect the gene function in other ways that need to be studied further. Accordingly, the biological effect of *KCNH7* on the efficacy of risperidone treatment in schizophrenia has been added to our study agenda.

This study has some limitations. First, the sample size was small. Therefore, further studies are needed to enroll more patients to confirm our present findings in this study. Second, the relatively sparse points selected in our study might have precluded the possibility of detecting some potential functional SNPs. Third, the biological function of rs77699177 was not sufficiently explored.

In this study, the SNPs in *KCNH7* showed significant associations with the treatment response to risperidone in 393 patients of Han Chinese ancestry. The results indicate the essential role of *KCNH7* in risperidone’s therapeutic efficacy. *KCNH7* might serve not only as a new functional marker for predicting the response to risperidone but also as a new drug target in the discovery of new antipsychotics. *KCNH2*, more commonly known as *hERG*, is primarily expressed in the brain and plays an essential role in cardiac action potential, and its mutations cause long QT syndrome (34). The *KCNH2*-related effect of risperidone would be limited because of its potential side effect on QT interval prolongation; however, *KCNH7* might not have similar side effects. Therefore, *KCNH7* could be an ideal target for designing new antipsychotics. Further studies are needed to determine whether risperidone can bind to these potassium channels. Moreover, studies on the difference in regulation and effects between *KCNH7* and *KCNH2* will help understand the function of the Kv channel in schizophrenia.

## Ethics Statement

The study was approved by the research ethical committees and institutional review boards of each local hospital. All procedures were conducted in accord with principles expressed in the Declaration of Helsinki. The study was registered for clinical trial number as ChiCTR-RNC-09000522 (http://www.chictr.org/).

## Author Contributions

WY and YH designed the study and provided the funds. XW wrote the manuscript and analyzed the data with YS. HY and ZH supervised this study.

## Funding

The study was funded by the National Key R&D Program of China (2016YFC1307000), National Natural Science Foundation of China (81571313 and 91432304), National High Technology Research and Development Program of China (2009AA022702), Beijing Nova Program Interdisciplinary Studies Cooperative Projects (Z161100004916038), Peking University Seed Fund for Medicine-Information Interdisciplinary Research Project (BMU20160597), the Fund for Fostering Young Scholars of Peking University Health Science Center (BMU2017PY030), Peking University Clinical Scientist Program (BMU2019LCKXJ012), supported by “the Fundamental Research Funds for the Central Universities”, and National Science and Technology Major Project for IND (investigational new drug) 2018ZX09201-014.

## Conflict on Interest Statement

The authors declare that the research was conducted in the absence of any commercial or financial relationships that could be construed as a potential conflict of interest.
