# Supplementary material for: Association Study of KCNH7 Polymorphisms and Individual Responses to Risperidone Treatment in Schizophrenia
Source: Front Psychiatry. 2019 Aug 30;10:633. doi: 10.3389/fpsyt.2019.00633 (PMC6728906; doi:10.3389/fpsyt.2019.00633)
Supplement: Supplementary file 1 [file DataSheet_1.docx]

***Supplementary*** ***Material***

**Association Study of *KCNH7* Polymorphisms and Individual Responses to Risperidone Treatment in Schizophrenia**

Xueping Wang, Ph.D., Yi Su, Hao Yan, Ph.D., Zhuo Huang, Ph.D., Yu Huang*, Ph.D., Weihua Yue*, M.D.

**Correspondence:**

*e-mail: Weihua Yue, dryue@bjmu.edu.cn or Yu Huang, hy@pku.edu.cn

**Supplementary Table 1. PCR and extension primer sequences from Sequenom SNP genotyping.**

| SNP | Position | 2nd-PCRP | 1st-PCRP | UEP_SEQ |
| --- | --- | --- | --- | --- |
| rs2241240 | chr2:162422815 | ACGTTGGATGGGGCACAGCGTAGATTGTTT | ACGTTGGATGGAGGTCAGGAAGCTTAAGTA | ACAGCGTAGATTGTTTAAAATA |
| rs77699177 | chr2:162476371 | ACGTTGGATGTTGCCTGGCATTACTATTGG | ACGTTGGATGTCAGGATAATGAGTTTTGC | CATTACTATTGGATAAGAATACCT |
| rs13404874 | chr2:162539839 | ACGTTGGATGCTTTTCTCTTTTAGTTTGGG | ACGTTGGATGCCATGTATCTCCTTTCATTG | AATCTTGCATAGCCTCC |
| rs16846992 | chr2:162611723 | ACGTTGGATGGAGCATGTTGTGAGTTAAACC | ACGTTGGATGGGAGAAAATGGGTGCTACTC | TGAGTTAAACCTGCAATTT |
| rs12991788 | chr2:162731734 | ACGTTGGATGCTATACTAAATTTAGGGTTGC | ACGTTGGATGTTCCTCTGCCCTCTATAAAG | AAATTTAGGGTTGCAGATAC |
| rs1017406 | chr2:162786897 | ACGTTGGATGCTTACCATCTGCTGTGCATC | ACGTTGGATGCCATGCTCTAAGGAATGCAG | CTTCGCTCAAGTGCT |

**Expression quantitative trait locus (eQTL) analysis about KCNH7**

In order to detect the eQTL effect of *KCNH7* gene, we searched the SNPs in Braineac Database (1) (<http://www.braineac.org/>). The database was consisted by 134 neuropathologically normal donors. Unfortunately, the SNPs information in Braineac Database were processed on Affymetrix Human Exon 1.0 ST arrays, so the intron SNPs analyzed in this study were not included in this database. Using this database, we examined eQTL data of *KCNH7* gene in different brain tissues and listed the SNPs information (Supplementary Table 2).

**Supplementary Table 2. The eQTL analyses for KCNH7 gene using the Braineac database.**

| **Tissue** | **rsID** | **Pos** | **Transcript ID** | **p-value** |
| --- | --- | --- | --- | --- |
| **CRBL** |  |  |  |  |
|  | rs918927 | chr2:162978918 | 2584258 | 2.20E-12 |
|  | rs2216897 | chr2:162979505 | 2584258 | 2.30E-12 |
|  | rs918928 | chr2:162978985 | 2584258 | 2.30E-12 |
|  | rs1863101 | chr2:162978887 | 2584258 | 2.30E-12 |
|  | rs918929 | chr2:162979087 | 2584258 | 2.30E-12 |
|  | rs13383228 | chr2:162977477 | 2584258 | 2.30E-12 |
|  | rs12052528 | chr2:162976938 | 2584258 | 2.30E-12 |
|  | rs11902505 | chr2:162975946 | 2584258 | 2.30E-12 |
|  | rs6432740 | chr2:162976417 | 2584258 | 2.30E-12 |
|  | rs918927 | chr2:162978918 | 2584287 | 6.20E-12 |
| **FCTX** |  |  |  |  |
|  | rs79820653 | chr2:162154167 | 2584303 | 2.20E-05 |
| **MEDU** |  |  |  |  |
|  | rs34193907 | chr2:163075852 | 2584283 | 4.60E-05 |
|  | rs62188206 | chr2:162488054 | 2584283 | 5.00E-04 |
| **OCTX** |  |  |  |  |
|  | rs12477167 | chr2:163052752 | 2584283 | 3.80E-05 |
|  | rs13016736 | chr2:162780891 | 2584283 | 4.90E-04 |
| **SNIG** |  |  |  |  |
|  | rs13015258 | chr2:162074215 | 2584302 | 1.70E-03 |
| **TCTX** |  |  |  |  |
|  | rs79084645 | chr2:163768240 | 2584299 | 8.50E-11 |
|  | rs78285421 | chr2:163768260 | 2584299 | 8.50E-11 |
|  | rs16848913 | chr2:163769814 | 2584299 | 1.10E-10 |
|  | rs9646769 | chr2:163772862 | 2584299 | 1.10E-10 |
|  | rs75000171 | chr2:163773652 | 2584299 | 1.10E-10 |
|  | rs9646769 | chr2:163772862 | 2584272 | 9.50E-10 |
|  | rs75000171 | chr2:163773652 | 2584272 | 9.50E-10 |
|  | rs16848913 | chr2:163769814 | 2584272 | 9.50E-10 |
|  | rs78285421 | chr2:163768260 | 2584272 | 9.90E-10 |
|  | rs79084645 | chr2:163768240 | 2584272 | 1.00E-09 |

Data from the Braineac database ([http://www.braineac.org](http://www.braineac.org/)), the expression Quantitative Trait Loci (eQTL) of KCNH7 gene in several brain tissues (CRBL: cerebellar cortex; FCTX: frontal cortex; MEDU: inferior olivary nucleus; OCTX: occipital cortex; SNIG: substantia nigra; TCTX: temporal cortex). Transcript ID: ID of exon-specific transcript probe sets. The eQTL from CRBL and TCTX were only listed the former 10 Loci.

All genomic locations are given as NCBI GRCh38 coordinates.

**Supplementary Table 3. The responder analysis for SNPs of *KCNH7* gene.**

| **SNP** | **Genotype** | **Response rate (%)** | | |
| --- | --- | --- | --- | --- |
|  |  | **2 weeks** | **4 weeks** | **6 weeks** |
| rs77699177 | CC | 12.26 | 40.11 | 65.74 |
|  | TC | 17.65 | 58.82 | 88.24 |
|  |  |  |  |  |
| rs2241240 | TT | 12.74 | 41.40 | 70.38 |
|  | CT+CC | 12.66 | 43.04 | 56.96 |
|  |  |  |  |  |
| rs13404874 | GG | 12.29 | 41.95 | 71.19 |
|  | AG | 11.51 | 40.29 | 62.59 |
|  | AA | 27.78 | 50.00 | 61.11 |
|  |  |  |  |  |
| rs16846992 | AA | 13.89 | 41.67 | 69.10 |
|  | GA | 6.38 | 40.43 | 63.83 |
|  | GG | 36.36 | 54.55 | 63.64 |
|  |  |  |  |  |
| rs12991788 | AA | 12.23 | 41.69 | 67.71 |
|  | GA+GG | 14.86 | 41.89 | 67.57 |
|  |  |  |  |  |
| rs1017406 | CC | 21.15 | 51.92 | 72.12 |
|  | CA | 9.42 | 36.65 | 63.87 |
|  | AA | 10.20 | 40.82 | 70.41 |

The continuous PANSS clinical ratings were dichotomized into two group according to 50% PANSS percentage change as the threshold value. The response rate for each genotype of different SNPs were calculated. The minor allele of rs2241240 and rs12991788 in this study were less than 4, thus they were incorporated into heterozygous genotype in the analyses.

**Figure S1. Gene expression pattern of Kv11 family in human tissues.**

Gene expression of *KCNH7*, *KCNH2*, and *KCNH6* in different human tissues. The expression data comes from https://www.ncbi.nlm.nih.gov/gene, the RNA-seq result performed by the HPA RNA-seq normal tissues project. RPKM, Reads Per Kilobase per Million mapped reads.

**Figure S2. Gene expression pattern of *KCNH7*, *KCNH2* and *KCNH6* range from embryonic development to adulthood.**

The images are derived from The HBT (Human Brain Transcriptome) project (2). <http://hbatlas.org/pages/hbtd>

**Figure S3. The eQTL effects of rs918927 and rs2216897 within *KCNH7* gene.**

The pictures are derived from the Braineac database. The gene expression analysis in ten brain tissues: TCTX: temporal cortex; FCTX: frontal cortex; OCTX: occipital cortex; CRBL: cerebellar cortex; MEDU: inferior olivary nucleus; PUTM: putamen; THAL: thalamus; HIPP: hippocampus; SNIG: substantia nigra; WHMT: intralobular white matter.

**Figure S4. The gene expression of *KCNH7*, *KCNH2* and *KCNH6* in define cell type.**

The pictures are derived from the AlzData (3) (http://www.alzdata.org). The gene expression data from single cell RNA-seq for endothelial cells, astrocytes, microglia, oligodendrocytes, oligodendrocyte precursor cells (OPCs) and neurons are displayed. This dataset (accession no. GSE67835) was originally from a human brain transcriptome study (4).

**Figure S5. Differential expression of *KCNH7* between control and schizophrenia**

The pictures are derived from the schizophrenia database (5) (SZDB, <http://www.szdb.org/index.html>).

**Supplementary References:**

1. Ramasamy A, Trabzuni D, Guelfi S, Varghese V, Smith C, Walker R, et al. Genetic variability in the regulation of gene expression in ten regions of the human brain. *Nat Neurosci* (2014) 17(10):1418-28. doi: 10.1038/nn.3801

2. Kang HJ, Kawasawa YI, Cheng F, Zhu Y, Xu X, Li M, et al. Spatio-temporal transcriptome of the human brain. *Nature* (2011) 478(7370):483-9. doi: 10.1038/nature10523

3. Xu M, Zhang DF, Luo R, Wu Y, Zhou H, Kong LL, et al. A systematic integrated analysis of brain expression profiles reveals YAP1 and other prioritized hub genes as important upstream regulators in Alzheimer's disease. *Alzheimers Dement* (2018) 14(2):215-29. doi: 10.1016/j.jalz.2017.08.012

4. Darmanis S, Sloan SA, Zhang Y, Enge M, Caneda C, Shuer LM, et al. A survey of human brain transcriptome diversity at the single cell level. *Proc Natl Acad Sci U S A* (2015) 112(23):7285-90. doi: 10.1073/pnas.1507125112

5. Wu Y, Yao YG, Luo XJ. SZDB: A Database for Schizophrenia Genetic Research. *Schizophr Bull* (2017) 43(2):459-71. doi: 10.1093/schbul/sbw102
