# Supplementary figures and images for: Association Study of KCNH7 Polymorphisms and Individual Responses to Risperidone Treatment in Schizophrenia
Source: Front Psychiatry. 2019 Aug 30;10:633. doi: 10.3389/fpsyt.2019.00633 (PMC6728906; doi:10.3389/fpsyt.2019.00633)

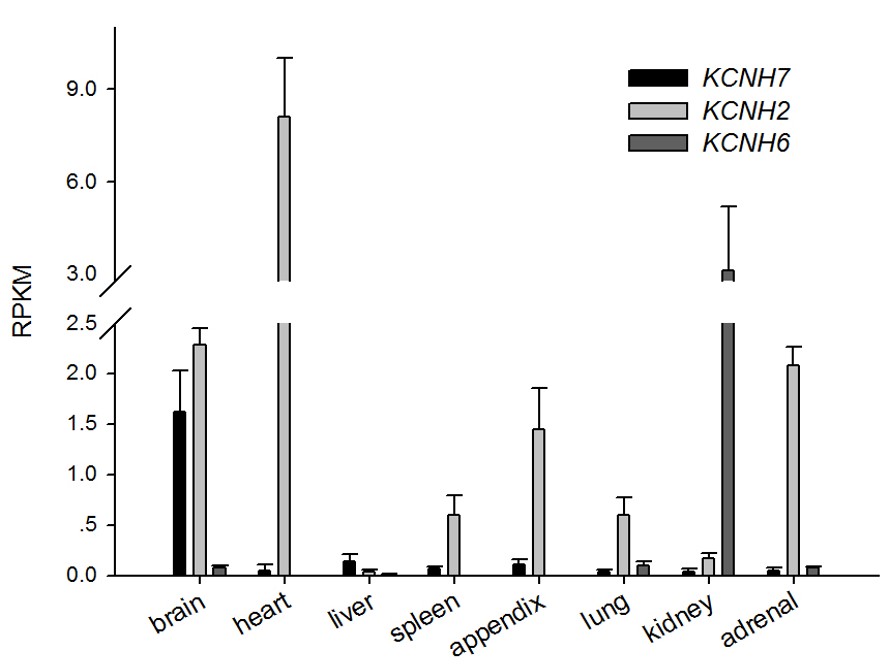

Supplement: Supplementary file 2 [file Image_1.jpeg]

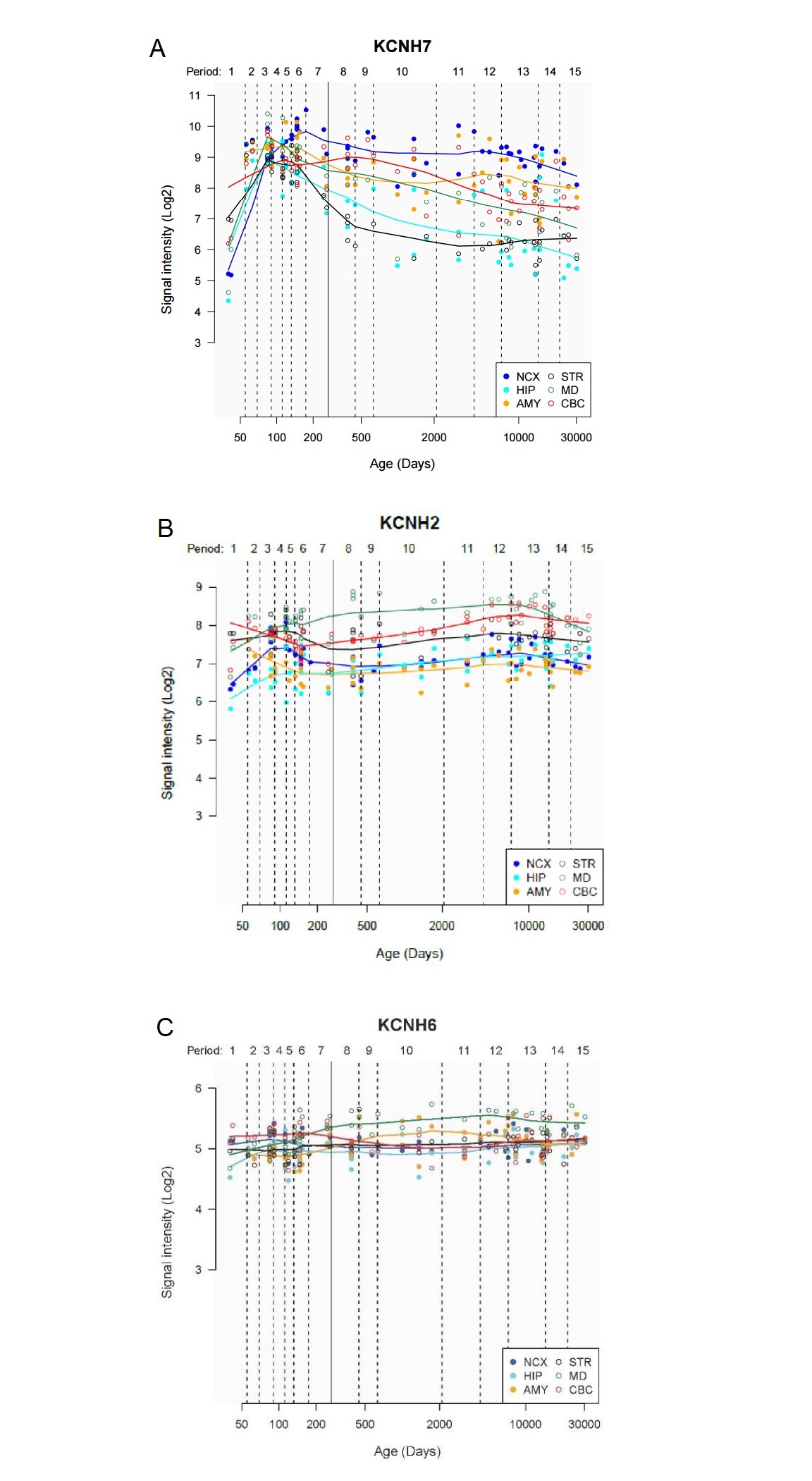

Supplement: Supplementary file 3 [file Image_2.jpeg]

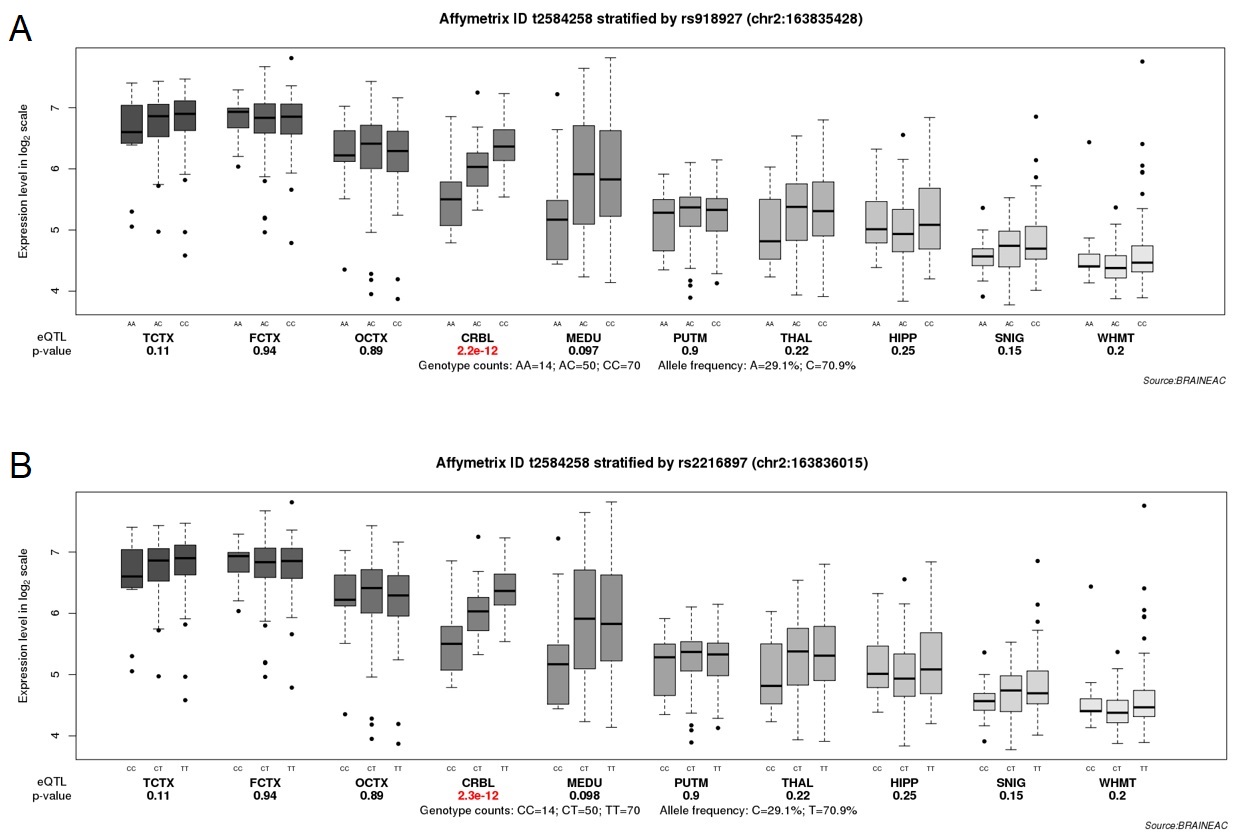

Supplement: Supplementary file 4 [file Image_3.jpeg]

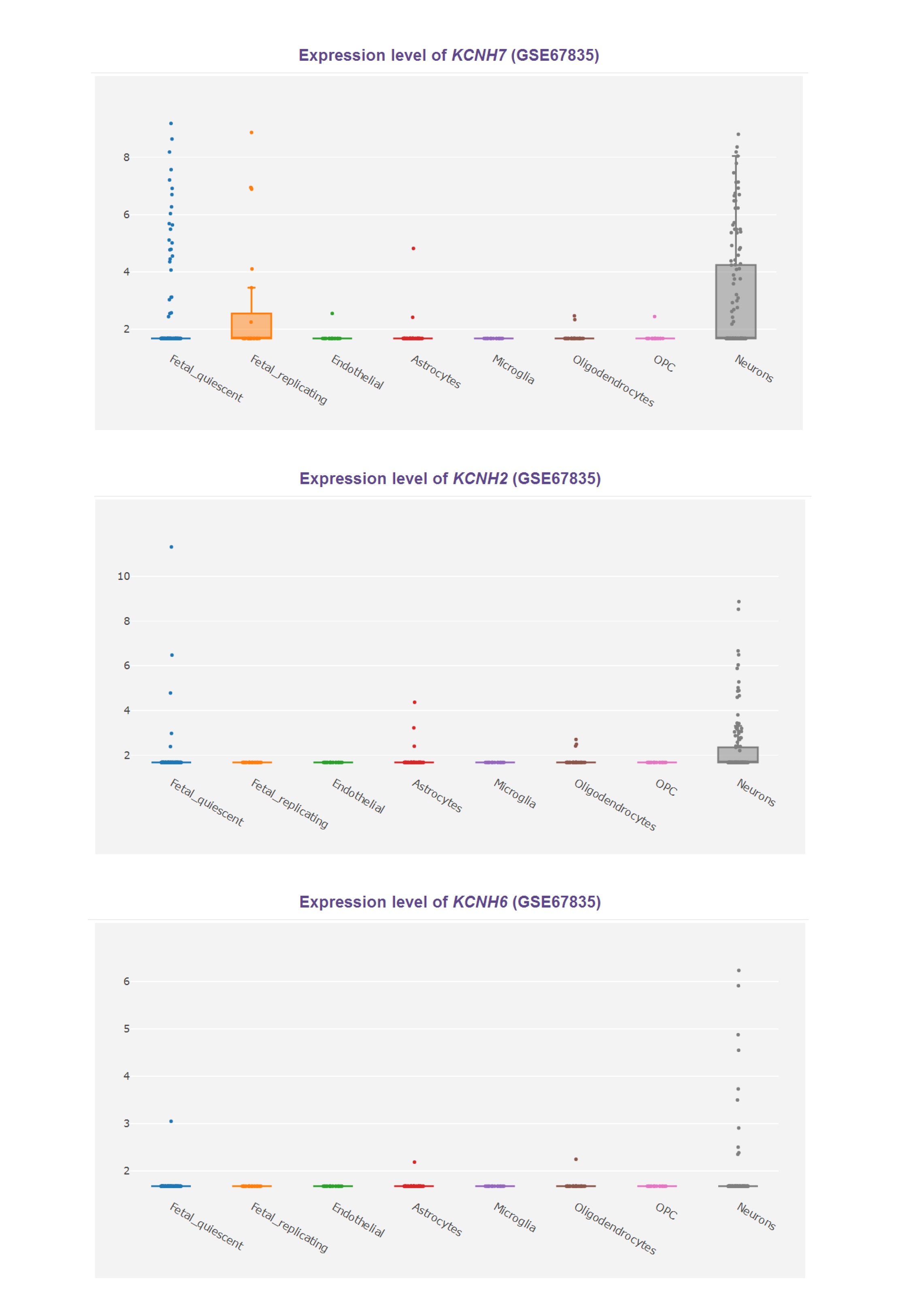

Supplement: Supplementary file 5 [file Image_4.jpeg]

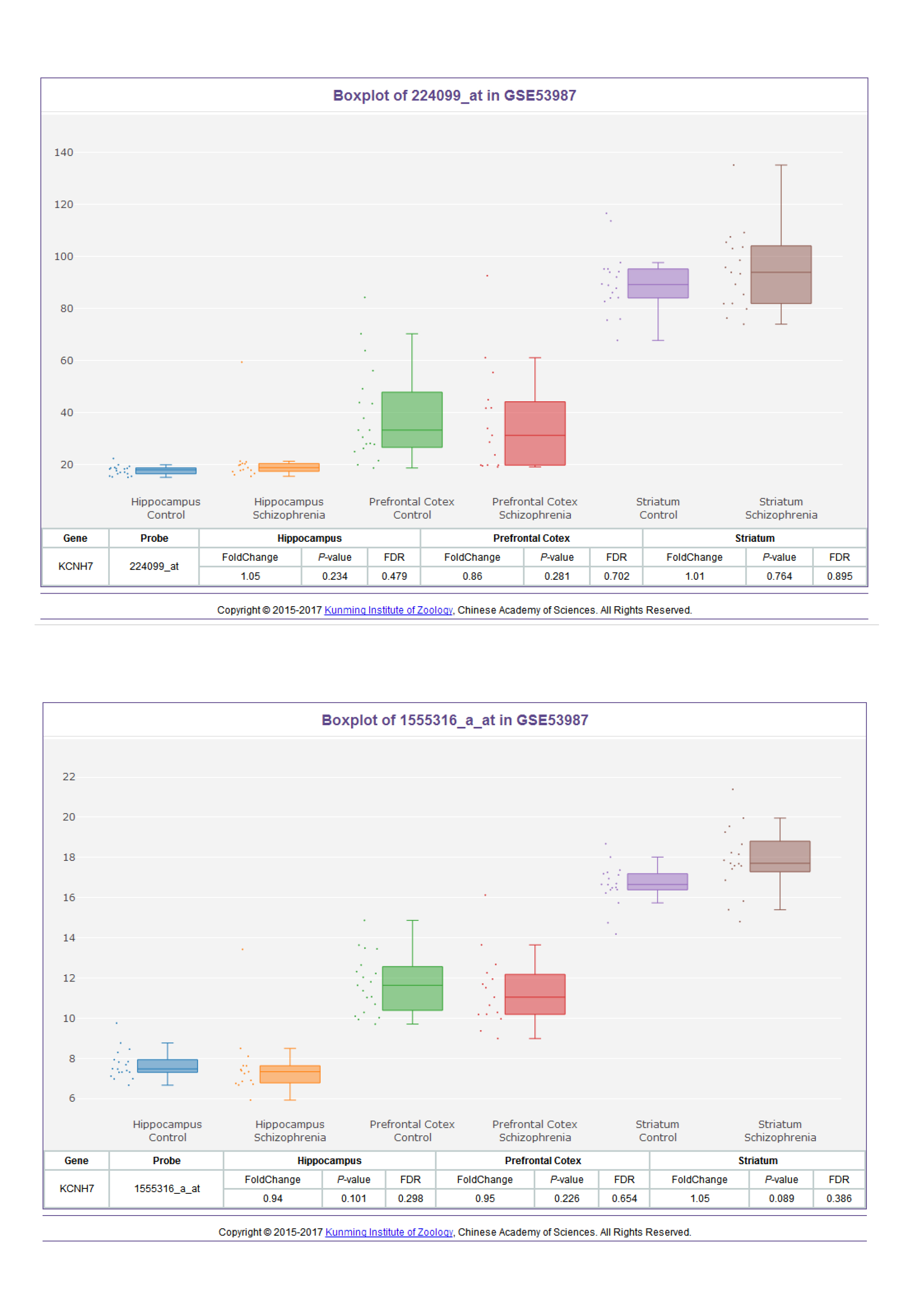

Supplement: Supplementary file 6 [file Image_5.jpeg]
